# New horizons in tumor microenvironment biology: challenges and opportunities

**DOI:** 10.1186/s12916-015-0278-7

**Published:** 2015-03-05

**Authors:** Fei Chen, Xueqian Zhuang, Liangyu Lin, Pengfei Yu, Ying Wang, Yufang Shi, Guohong Hu, Yu Sun

**Affiliations:** Key Laboratory of Stem Cell Biology, Institute of Health Sciences, Shanghai Institutes for Biological Sciences, Chinese Academy of Sciences/Shanghai Jiaotong University School of Medicine, Shanghai, 200031 China; Soochow Institutes for Translational Medicine, Soochow University, Suzhou, 215123 China; VA Seattle Medical Center, Seattle, WA 98108 USA; Department of Medicine, University of Washington, Seattle, WA 98195 USA; Institute of Health Sciences, Shanghai Institutes for Biological Sciences (SIBS), Chinese Academy of Sciences (CAS) and Shanghai Jiaotong University School of Medicine (SJTUSM), 320 Yue Yang Road, Biological Research Building A, Shanghai, 200031 China

**Keywords:** Acquired resistance, Clinical oncology, Combination therapy, Distant metastasis, Immunomodulation, Targeting strategy, Therapeutic intervention, Translational medicine, Tumor microenvironment

## Abstract

The tumor microenvironment (TME) is being increasingly recognized as a key factor in multiple stages of disease progression, particularly local resistance, immune-escaping, and distant metastasis, thereby substantially impacting the future development of frontline interventions in clinical oncology. An appropriate understanding of the TME promotes evaluation and selection of candidate agents to control malignancies at both the primary sites as well as the metastatic settings. This review presents a timely outline of research advances in TME biology and highlights the prospect of targeting the TME as a critical strategy to overcome acquired resistance, prevent metastasis, and improve therapeutic efficacy. As benign cells in TME niches actively modulate response of cancer cells to a broad range of standard chemotherapies and targeted agents, cancer-oriented therapeutics should be combined with TME-targeting treatments to achieve optimal clinical outcomes. Overall, a body of updated information is delivered to summarize recently emerging and rapidly progressing aspects of TME studies, and to provide a significant guideline for prospective development of personalized medicine, with the long term aim of providing a cure for cancer patients.

## Introduction

Cancer is a systemic disease, and it is not a solo production but rather an ensemble performance [1]. Cancer cells act as the leading devil, which is supported by a diverse cast of benign cells in the surrounding milieu that actively facilitates the malignant progression in a three-dimensional structure. Even under therapeutic conditions, resistant cancer clones frequently emerge and show complex dynamics with spatial and temporal heterogeneity, implying distinct mechanisms of resistance operative at different sites depending on treatment selection pressure [2,3]. The disease is usually initiated as a result of the stepwise accumulation of genetic and epigenetic changes in the epithelial compartment; however, increasing evidence indicates that the tumor microenvironment (TME) can dictate aberrant tissue function and play a critical role in the subsequent development of more advanced and refractory malignancies [4]. Particularly, inappropriate activation of the stroma, including those provoked by the therapeutics, immunomodulation mediated by certain TME cell lineages, and distant metastasis induced by the TME components, can potentiate and accelerate tumor progression towards a high rate of disease mortality [5].

Physiologically, the stroma in healthy individuals is a physical barrier against tumorigenesis; however, neoplastic cells elicit various changes to convert the adjacent TME into a pathological entity. The orchestration of such an event implicates migration of stromal cells, remodeling of matrix, and expansion of vasculature [6]. Regional differences under selective pressures, including acidity and hypoxia in the neoplasia, drastically influence its progression, as do distinct environmental factors select for mutations that engender survival and repopulation of cancer cells, eventually creating tumor heterogeneity and causing treatment difficulty [7]. In this review, we define the biological landscapes of neoplastic cell extrinsic environment, branded the TME, discuss therapeutic resistance that engages multiple stromal cell types, and present clinical challenges lying ahead which may be well taken by implementing effective strategies to deliver personalized cancer therapy.

## The TME is a pathologically active niche that shapes tumor evolution

The structurally and functionally essential elements in the stroma of a typical TME include fibroblasts, myofibroblasts, neuroendocrine cells, adipose cells, immune and inflammatory cells, the blood and lymphatic vascular networks, and the extracellular matrix (ECM). The naive stroma is a critical compartment in maintaining physiological homeostasis of normal tissue, and recent studies strengthened the concept that some stromal components have anticancer activities by regulating immunosuppression and restraining carcinogenesis, which is particularly the case of pancreatic ductal adenocarcinoma [8,9]. The ability of the stroma to suppress carcinogenesis apparently correlates with organismal survival and contribute to longevity. However, once transformed to a tumor-associated neighbor by various stimuli, the stroma-derived effect turns to be adverse and can significantly promote cancer progression. Under such conditions, the stromal cells co-evolve with the cancer cells by being frequently educated, coopted, or modified by the latter to synthesize a wide variety of cytokines, chemokines, growth factors, and proteinases, together dramatically accelerating disease progression [6]. Thus, normal stroma possesses an inherent plasticity to respond rapidly to neoplastic situations, and act in concert with the adjacent epithelium in eliciting the emergence of “reactive stroma”. The active stroma of solid tumors is not only composed of carcinoma-associated fibroblasts (CAFs) and myofibroblasts, but characterized with remodeled matrix, reprogrammed metabolism, activated transcription, and altered synthesis of repair-associated proteins [10-12]. Further, the physical or biological protection provided by the stromal part of the TME limits the effective delivery of anticancer agents to tumor foci and represents a favorable milieu that allows cancer cells to circumvent programmed cell death triggered by cytotoxicity and to develop acquired resistance as a preliminary step towards more malignant phenotypes.

Progression of organ-specific tumors is also reliant on infiltration of immune cells and occurrence of angiogenesis, which generates a stash for cancer stem cells (CSCs) and provides a complex signaling environment. CSCs, also known as tumor-initiating cells, have been intensively explored within the recent decade. Many tumor types involve CSCs in the TME milieu, which are characterized with the potential to cause resistance against various cytotoxicities due to intrinsic mechanisms, including genetic changes and epigenetic alterations. Both CAFs and CSCs are implicated in the TME-mediated signaling to remodel cancer cells; for instance, CAFs express high levels of extracellular factors including chemokine CXC motif ligand (CXCL)12, chemokine CC motif ligand (CCL)2, CCL8, and insulin-like growth factor binding protein 7, thereby forming an inflammatory niche [13-15]. Further, CSCs are highly responsive to immune modulation, and an immune signature is present in human prostate CD133^+^ CSCs, including interleukin (IL)-6 and interferon-γ receptor 1 [16].

Under *in vivo* conditions both the innate and adaptive immune systems influence homeostasis, in particular the recruitment of immune cells into the tumor-adjacent milieu is active and forms distinct immune contextures, thereby exerting profound impacts on clinical outcome. For example, T cell activation involves both positive and negative checkpoint signals to finely tune responses to prevent excessive pathological changes [17,18]. The myeloid-derived suppressor cell (MDSC) population which encompasses immature dendritic cells, neutrophils, monocytes, and early myeloid progenitors implicates tumor-initiated endocrine signaling to the immune system through multiple chemokines such as granulocyte-macrophage colony stimulating factor [19,20]. Some immunosuppressive myeloid lineages not only inhibit adaptive immunity, but promote angiogenesis through secretion of soluble molecules like vascular endothelial growth factor (VEGF) A, basic fibroblast growth factor (FGF), and transforming growth factor β (TGF-β) [21]. Independent of T cell activities, B cells are able to facilitate disease progression by fostering pro-tumoral inflammation [22]. Furthermore, type II tumor-associated macrophages (TAMs) drastically affect tumorigenesis, angiogenesis, and intravasation, and can prevent immune attack by natural killer (NK) and T cells during tumor development and after recovery from chemo- and/or immunotherapy [23].

In addition to many *in vitro* studies that prove the complex role of the TME cell lineages, experimental animal models with genetically modified stroma further presented convincing data of the biological importance of the TME. Genetic alterations in stromal fibroblasts caused pathologies in the adjacent glandular epithelium, as demonstrated by FGF10 overexpression in a tissue recombination model and TGF-β type II receptor conditional elimination in transgenic mice [24,25]. Thus, signaling activities of a single factor in fibroblasts can modulate the oncogenic potential of nearby epithelia in selected tissues. A new study even reported that p62 deficiency in the prostate stroma results in deregulation of cellular redox through an mTORC1/c-Myc pathway of glucose and amino acid metabolism, and upregulation of stromal IL-6 through c-Myc inactivation induces a hyper-inflammatory phenotype [11]. Simultaneously, an autocrine pathway promotes TGF-β and the induction of a CAF phenotype, which further increases epithelial invasion and tumorigenesis. As metabolic reprogramming of the stroma can decisively influence the tumorigenic potential of the epithelial compartment, this TME property is increasingly recognized as a potent candidate of therapeutic targets.

## The TME acts as a dominant force to modify treatment responses

Therapeutic resistance remains a major problem in clinical oncology. In addition to fueling *de novo* tumorigenesis, a permissive TME modifies treatment responses by affecting cell sensitivity to anticancer agents. The TME-induced resistance to interventions applied for multiple tumor types, as well as its magnitude, varies depending on the cancer cells, stroma properties, and therapeutic regimens. Further, drug resistance mediated by the TME is not limited to classical agents such as those administered in genotoxic chemotherapies; rather, it covers diverse pharmaceuticals including targeted agents [26]. Recent studies intensively evaluated the functional roles of TME in protecting acute myeloid leukemia cells or chronic lymphocytic leukemia cells against alkylating agents, anthracyclines, imatinib and nucleoside analogues, mutant Janus kinase 2 (JAK2) cells against JAK inhibitors including tofacitinib and ruxolitinib, solid tumors such as lung, colorectal, and head and neck malignancies against erlotinib and cetuximab, as well as, more recently, melanoma against RAF inhibitors like vemurafenib [27-29]. TME-mediated resistance can be initiated by multiple cell lineages and structural components in the stroma, including but not limited to fibroblasts, endothelial cells, pericytes, smooth muscle cells, neutrophils, macrophages, integrins, fibronectins, and collagens [26,30].

Although numerous reports elaborated the biological role of TME-derived factors in tumor growth or metastasis, relatively few have delineated the impact of an agent-activated TME to the therapeutic outcome. Recent studies using targeted drugs or conventional chemotherapeutics have filled the gaps to show that treatment-induced alterations to the microenvironment can generate a protective niche or shielding reservoir for the remnant cancer cell population, which is termed minimal residual disease as the occult site to prime tumor relapse [31]. Particularly, resistance to chemotherapy frequently results from cell extrinsic factors such as cytokines, growth factors, and even proteases derived from a TME that is structurally and functionally modified by drug-induced cytotoxicity [32-34]. In such cases, CSCs represent the potential source of eventual tumor relapse following therapy, which are typically therapy-resistant due to decreased oxidative stress response, increased genomic stability, and expression of multiple drug resistance transporters [35].

TME-exerted protection for cancer cells apply to multiple therapeutic situations. Upon melanoma treatment by mitogen activated protein kinase (MAPK) pathway inhibitors, TAMs expand and release tumor necrosis factor (TNF)-α as a crucial growth factor that provides resistance to the targeted therapy through the microphthalmia transcription factor [36]. Inhibiting TNF-α signaling with IκB kinase inhibitors profoundly enhanced the efficacy of MAPK pathway suppression by targeting not only the melanoma cells but the microenvironment. In experiments using doxorubicin to treat the well-established Eμ-Myc model of Burkitt’s lymphoma, surviving metastatic cancer cells were localized in the thymus [37]. Damage response analyses in different lymphoid tissues and the derived cell types revealed that thymic endothelial cells selectively secreted IL-6 and Timp-1 as prosurvival factors, both significantly enhancing resistance of lymphoma. Interestingly, inhibition of these factors or the upstream signaling pathway mediated by p38 mitogen-activated protein kinase (p38MAPK) increased the subsequent chemotherapeutic efficacy. Similarly, a genome-wide study of transcriptional responses of prostate stromal cells to genotoxic stress uncovered a spectrum of soluble proteins topped by WNT16B, a novel TME effector generated by the DNA damage secretory program [38]. Expression of WNT16B is regulated by NF-kB after DNA damage and subsequently activates the canonical Wnt pathway in adjacent cancer epithelial cells, thus markedly attenuating the effects of cytotoxic chemotherapy [39]. Further, chemotherapeutic agents to breast cancer trigger a parallel stromal reaction represented by TNF-α production in endothelial cells, which heightens the CXCL1/2 expression of cancer cells via the NF-kB complex, eventually amplifying a CXCL1/2-S100A8/9 loop and inducing chemoresistance [40]. Collectively, the results present a mechanism by which genotoxic therapies or targeted agents given in a cyclical manner can enhance subsequent treatment resistance through cell non-autonomous programs that are attributed to the “treatment-activated TME” [26] (Table 1).

**Table 1:** **Some anticancer treatments are subject to acquired resistance provoked by stromal factors derived from the disease-supporting TME**

**Therapeutics**	**Cancer type**	**Targeting mechanism**	**Resistance mechanism**	**Reference**
Doxorubicin	Multiple myeloma	Generate DNA intercalation; inhibit topoisomerase II	Stroma-induced resistance	[41]
PD184352	BRAF-mutant melanoma	Block MAPK pathway as an ATP non-competitive MEK1/2 inhibitor	Macrophage-derived TNF-α promotes microphthalmia transcription factor expression in *Braf* ^V600E^ melanoma cells, reducing caspase-3 cleavage under anoikis conditions	[36]
External beam radiation therapy	Anaplastic thyroid cancer	Generate DNA intercalation; inhibit topoisomerase II	Stroma-induced resistance; plays an important role in mortality of thyroid cancer	[42]
Mitoxantrone and docetaxel	Prostate cancer	Interrupt microtubule depolymerisation/disassembly; generates DNA strand breaks, inhibit topoisomerase II	Stroma-induced resistance through secretion of multiple soluble factors, with WNT16B as a major contributor	[39]
Doxorubicin	Burkitt’s lymphoma	Generate DNA intercalation; inhibit topoisomerase II	Stroma-induced resistance; paracrine factors including IL-6 and Timp-1 from thymic endothelial cells in the tumor microenvironment modulate lymphoma cell survival following chemotherapy	[37]
Doxorubicin and cyclophosphamide (AC regimen)	Breast cancer	Generate DNA intercalation; inhibits topoisomerase II and interferes with DNA replication	Stroma-induced resistance; chemotherapeutic agents trigger a stromal reaction leading to TNF-α production by endothelial and other stromal cells	[40]
Vemurafenib (PLX4032)	BRAF^V600E^-mutant melanoma; BRAF-mutant colorectal cancer and glioblastoma	Interrupts the B-Raf/MEK step on the B-Raf/MEK/ERK pathway	Stroma-induced resistance; resistance to RAF inhibitors is induced by hepatocyte growth factor secreted from tumor-adjacent stromal cells	[43,44]
Ruxolitinib (INCB018424)	JAK2^V617F^-mutant myeloproliferative disorders and high-risk myelofibrosis (a type of bone marrow cancer)	Inhibits Janus kinase inhibitor with selectivity for subtypes JAK1 and JAK2 of this enzyme	Stroma-induced resistance; humoral factors secreted by stromal cells protect myeloproliferative neoplasms clones against JAK2 inhibitor therapy	[45]
Erlotinib and gefitinib	Metastatic lung, colorectal, pancreatic, or head and neck cancers	Inhibits the epidermal growth factor receptor (EGFR), can stimulate apoptosis and differentiation of cancer cell that lack EGFR	Substantial stroma-induced resistance; clinical responses to EGFR tyrosine kinase inhibitors and monoclonal antibodies are now tempered by the increasing number of *de novo* and acquired resistance mechanisms, the latter contributed by stroma	[27]
Afatinib	Metastatic non-small cell lung cancer, breast cancer, and other EGFR/Her2-driven cancers	Irreversibly inhibits EGFR and HER2 kinases	Stromal expression of fibroblast growth factor (FGF) 2 and the FGFR1 is upregulated, allowing survival of afatinib-resistant cancer cells	[46]

Although dominant anticancer regimens, including chemotherapy and targeted therapy, provide major options for cancer patients, so far, mounting data pinpoints to an intricate link between epithelial-mesenchymal transition (EMT) and therapeutic resistance. Gain of function as resistance for cancer cells can be regulated by diverse mechanisms, and it may arise as a direct consequence of EMT triggered by a large array of the TME-derived molecules through activation of intracellular networks that cover hepatocyte growth factor/c-met, epidermal growth factor (EGF)/EGF receptor (EGFR), Wnt/beta-catenin axes, and several cytokine/chemokine-mediated pathways such as TGF-β/Smad signaling [47-51]. In this regard, most treatment-resistant cancers harbor a subgroup of cells with stem-like or mesenchymal features that are resistant to cancer therapies [52].

TME-conferred resistance is not limited to solid tumors. A new study of leukemia identified a therapy-induced niche in the bone marrow, which empowers the resident leukemia propagating cells (LPCs) to survive with antiapoptotic properties [53]. Upon treatment with cytarabine and/or daunorubicin, the first-line chemotherapeutic agents for acute lymphoblastic leukemia patients, a protective TME was formed within the bone marrow. The niche was morphologically volatile and changed dynamically, beginning as transient Nestin + cells, maturing by switching to alpha small muscle actin cells, and ending as fiber residues. Emergence of such an evolving TME significantly contributes to treatment failure and precludes complete remission. Given that genetic or epigenetic reprogramming of niche-resident LPCs may occur to generate refractory subclones upon exposure to clinical treatments [54], the study highlights that future therapeutic strategies should be adjusted to prevent the arising of an early protective TME. Given a scenario of agents that target various TME cell lineages, one can envisage that combinatorial therapies provide an effective solution which both confines cancer cell progression and suppresses TME-associated activities.

## Tumor promotion by mesenchymal stem cells (MSCs) and regulatory T cells (Tregs) in the TME

Throughout the course of tumor evolution, a vast group of host cells, ranging from fibroblasts to macrophages, sustain a supportive TME for disease progression, specifically by interfering immunosurveillance against cancer cells [55]. Among these disease-favorable stromal cells, several subpopulations are virtually bone marrow-derived cells (BMDCs) and frequently implicated in tumor expansion via homing to the primary site as active components of the local TME. Being a typical representative of BMDCs but still keeping differentiation potential, MSCs mainly derive from the bone marrow but are indeed resident in virtually all organs and mature tissues, receiving much interest in recent years particularly in cancer biology. In contrast to TAMs, which compose a terminal lineage, MSCs remain primitive and can generate adipocytes, pericytes, chondrocytes, neurons, osteocytes, and mainstay stromal cells, including fibroblasts and endothelial cells, and can also transdifferentiate into both ectodermal and endodermal cells, thereby displaying a high plasticity and contributing to tissue regeneration [56-59]. MSCs migrate towards the tumor site and become a major component of tumor-adjacent stroma. Approximately 20% of CAFs originate from bone marrow and derive from MSCs, as demonstrated by studies using mouse models of inflammation-induced tumors [60]. Tumors employ various strategies to recruit MSCs and chemokines are the most reported; for instance, breast tumors secret monocyte chemotactic protein-1 to stimulate the migration of MSCs, while prostate tumors release CXCL16 to attract MSCs via binding to the CXCR receptor on these cells [43,61]. Once relocated to the tumor site, MSCs actively communicate with several cell types, including cancer cells and nearby immune cells, thus being biologically involved in the regulation of tumor development. Specifically, MSCs have intrinsic clinical value and hold potential for therapeutic use in stem cell-based cancer therapy as a vehicle to deliver gene products to targeted sites [62-64].

MSCs are capable of modulating immune status; however, the immunoregulatory function of MSCs is not intrinsic but depends on their cytokine milieu [65]. MSCs isolated from spontaneous lymphomas have a strikingly high expression of CCL2 compared with bone marrow-derived MSCs (BM-MSCs), and promote tumor growth by recruiting type 2 like TAMs to tumor site, a phenomenon that can be mimicked by treating BM-MSCs with tumor necrosis factor alpha (TNF-α) [66]. Combination treatment of MSCs with interferon gamma (IFN-γ) and TNF-α would dramatically increase the expression of several chemokines and inducible nitric oxide synthase (iNOS), a key immune suppressive molecule [67]. Further, the immunosuppressive effect of MSCs induced by IFN-γ and TNF-α can be dramatically enhanced by IL-17, which enhances mRNA stability by modulating the protein level of ARE/poly(U)-binding/degradation factor 1, a well-known factor that promotes mRNA decay [68]. T cell migration is driven by chemokines into proximity with MSCs, where T cell responsiveness is suppressed by nitric oxide (NO). The MSC-mediated immunosuppression may interfere with the anti-tumor immunity and help the tumor escape immunological surveillance. Interestingly, MSCs derived from p53-deficient mice express more iNOS and exhibited greater immunosuppressive capacity in the presence of inflammatory cytokines. When inoculated with B16F0 melanoma in mice, p53-deficient MSCs resulted in tumors larger than those harboring wild type MSCs, and such a tumor promoting effect could be abolished by administration of the iNOS inhibitor, S-methylisothiourea [69]. However, information collected from studies of the murine system may not be directly extended to humans because human MSCs utilized indoleamine 2,3-dioxygenase (IDO) instead of iNOS to suppress immune response [70]. Therefore, a recent study employed a humanized MSC system, which allows mouse iNOS promoter-driven IDO expression to be activated by inflammatory cytokines similar to the human IDO promoter [71]. Interestingly, humanized MSCs reduced the tumor-infiltrating CD8^+^ T cells and B cells when co-injected with tumor cells in mice, thus promoting tumor growth, highlighting the important interaction between MSCs and other components of the TME as well as the possibility of restoring tumor immunity in humans by therapeutic targeting IDO activity. Tumor-resident MSCs seem to be pathologically educated to favor tumor growth; inflammatory cytokines may act as a major driver for the change of local microenvironments, but other factors are also likely to be implicated in TME-exerted modifications. As supporting evidence, several studies demonstrated that exosomes derived from BM-MSCs can induce progenitor cells to undergo mesenchymal-to-epithelial transition (MET), indicating active message delivery between cancer cells and the bone marrow TME [72,73].

Of note, growth factors and cytokines released by MSCs invoke proliferative signaling of cancer cells and protect them against cell death, a function that can be exerted passively. Chemotherapy to leukemia elicits resistance by rebuilding an microenvironmental niche that allows cancer-propagating cells to evade apoptosis, and MSCs generate replatable mesenspheres and express CD29, CD51, and chemokine receptor CCR1 [47]. In ovarian cancer, MSC secretions promote phosphatidylinositol 3-kinase (PI3K)/Akt signaling and the X-linked inhibitor of apoptosis protein phosphorylation, inducing carboplatin-specific resistance through trogocytosis [74]. Interestingly, MSCs can also release two distinct polyunsaturated fatty acids, 12-oxo-5,8,10-heptadecatrienoic acid and hexadeca-4,7,10,13-tetraenoic acid, which are in minute quantities but induce resistance to a broad spectrum of chemotherapeutic agents, particularly platinum analogs [75].

The contribution of MSCs to tumor progression and resistance is well established, while the MSC-mediated Tregs expansion and immunosuppression has recently attracted increasing interest. In particular, human leukocyte antigen-G5 secreted by MSCs stimulates FoxP3^+^ CD25^Hi^ CD4^+^ Tregs proliferation and maintains the immunosuppressive activity by reducing T lymphocytes and NK functions for an extended period upon co-culture *in vitro* [76,77]. Tregs maintain immune tolerance and prevent inflammation by restraining the activity of cytotoxic T cells and the proliferation of effector T cells, correlating with poor prognosis in cancer patients. Depletion of Tregs inhibits the progression of breast cancer, leukemia, myeloma, fibrosarcoma, colon adenocarcinoma, and lung cancer, while primary tumor infiltration by Tregs promotes the metastatic potential [55].

## The TME is an essential determinant of the metastatic cascade

Metastasis accounts for approximately 90% of overall mortality among solid tumor patients [78]. The metastatic journey of cancer cells from original site to distant organs comprises several distinct stages, including local invasion, intravasation, circulationary survival, extravasation, and ectopic recolonization. Tumors not only preferentially select proclivity sites for metastasis, but exhibit variable dormancy length in temporary course [31], both as important facets to be considered for improved drug design and treatment strategy to thwart disease exacerbation at each individual stage. A supporting TME allows stromal cells to co-evolve with cancer cells, promoting the initial dissemination and subsequent invasion at the primary site and creating a permissive niche at the distant location. The microenvironments in metastatic lesions differ prominently from those of primary foci, and the formation of a receptive TME before the arrival of disseminated tumor cells enhances metastatic efficiency, substantiating the ‘seed and soil’ hypothesis raised by Paget in the 19^th^ century [79,80].

Local invasion is the physical entry of cancer cells resident within a well-confined primary tumor into the surrounding stroma. Cancer cells first breach the basement membrane, a specialized ECM structure in the TME, by co-opting the EMT program, which allows dissolution of tight junctions, loss of cell polarity, and acquisition of multiple mesenchymal attributes [81]. Stromal cells further enhance the aggressive behaviors of cancer cells through various types of signaling. For instance, breast cancer invasiveness can be stimulated by IL-6 secreted from adipocytes or promoted through EGFR-mediated signaling upon activation by TAMs that are subject to CD4^+^ T-lymphocyte instigation in the local microenvironment [78]. Thus, TME at primary site increases tumor dispersion via paracrine signals by generating a chemotactic relay system, a case that can be further exemplified by CXCL14 secreted by CAFs, or EGF and CXCL5 released by tumor-associated dendritic cells in prostate and lung tumors, respectively [82,83]. In addition, TME-associated hypoxia or inflammation causes tumor dissemination through multiple mechanisms, including the NO-dependent VEGF upregulation mediated by hypoxia-inducible factor 1α in endothelial cells [84] and hypoxia-recruited infiltration of BMDCs including MDSCs and NKs into secondary organs [85], each case remarkably promoting pre-metastatic dissemination in the primary organ.

Intravasation is a critical step that allows cancer cells to cross pericyte and endothelial cell barriers before they gain access to other organs [86,87]. For example, CDC42-mediated expression of integrin β1 supports the interplay of lung cancer cells with endothelium and thus promotes transendothelial migration, while TGF-β enhances breast cancer intravasation by increasing cancer cell penetration through microvessel walls [87,88]. Conversely, the transcriptional modulator amino-terminal enhancer of split blocks intravasation in colon cancer through Notch-dependent mechanisms [86]. Resembling the stromal lineages, cancer cells can also enhance vasculature permeability, particularly at the site of extravasation where normal endothelial cells are tightly organized, to gain transendothelial entrance by secreting factors including angiopoietin 1, angiopoietin-like 4, cytochrome c oxidase subunit 2, epiregulin, matrix metalloproteinase (MMP)-1/2/3/10, TGF-β, and VEGF in the case of lung or brain carcinoma [31].

Either at primary sites or in vasculature vessels, cancer cells can release microvesicles or soluble factors to adapt incipient metastatic sites into ‘pre-metastatic niches’; for example, systemic factors attract bone marrow-derived macrophages and hematopoietic progenitor cells that are accompanied by CAFs and endothelial cells to remodel tissue and eventually cause lung metastasis [89]. However, metastasis-incompetent cancer cells can foster a metastasis-compatible TME by secreting extracellular factors including thrombospondin 1 to promote niche formation at metastatic sites [90]. Successful seeding of cancer cells at secondary organs is just a prerequisite, and the TME at the secondary site may actually restrain ectopic cell survival and expansion as illustrated by neutrophil-mediated killing of cancer cells or thrombospondin 1 secretion by bone marrow-derived Gr1^+^ cells [90,91]. Interestingly, cancer cells can manage to evade initial cell-eliminating defense activities at distant sites and enter dormancy as micrometastases for a certain period before evident expansion or disease relapse. Tumor dormancy is regulated by several mechanisms, driven partially by the TME, including cellular dormancy (cells arrested in G_0_), tumor mass dormancy (proliferation refrained by apoptosis), or immune dormancy (an equilibrium maintained by immunosurveillance) [92]. Unfortunately, tumors are prone to be awakened by various stimuli such as acquired mutations arising from of cancer cell genomic instability, which allow them to exit dormancy for resumed metastatic progression, while more events of tumor awakening and distant outgrowth are driven by the TME constituents. A novel mechanism of triple-negative breast cancer metastasis was recently delineated, and involves the TME factors as peripheral signals, including EGF and insulin-like growth factor-I (IGF-I), at distant indolent tumor sites [93]. Bioavailability of EGF and IGF-I increases the expression of transcription factors associated with pluripotency, proliferation, and phenotypic transition, whereas combinatorial therapy to target EGF and IGF-I signaling prevents metastatic growth, suggesting that plasticity and recurrence rates can be dictated by host systemic factors and offer remarkable therapeutic potential for triple-negative breast cancer patients.

Micro RNAs (miRNAs) are circulated in cancer patient serum and can serve as important biomarkers for many cancer types [94]. New studies presented mechanistic evidence that some miRNAs directly regulate metastasis by mediating tumor–TME interactions. Particularly, miR-210 is released from metastatic breast cancer cells via nSMase2-dependent exosomal secretion, which once transported to endothelial cells can enhance cell migration and capillary formation, thereby enhancing angiogenesis and metastasis [95]. The miRNAs can also be transmitted from stroma cells to cancer cells as exemplified by microvesicle-delivered miR-223, which is highly expressed in IL-4-activated TAMs but not in breast cancer cells and which, upon transmission from TAMs to cocultured cancer cells, promotes tumor invasion and metastasis [96]. The transmission of miRNAs between different cell types provides an additional mechanism of TME-regulated metastasis.

**Figure 1:**
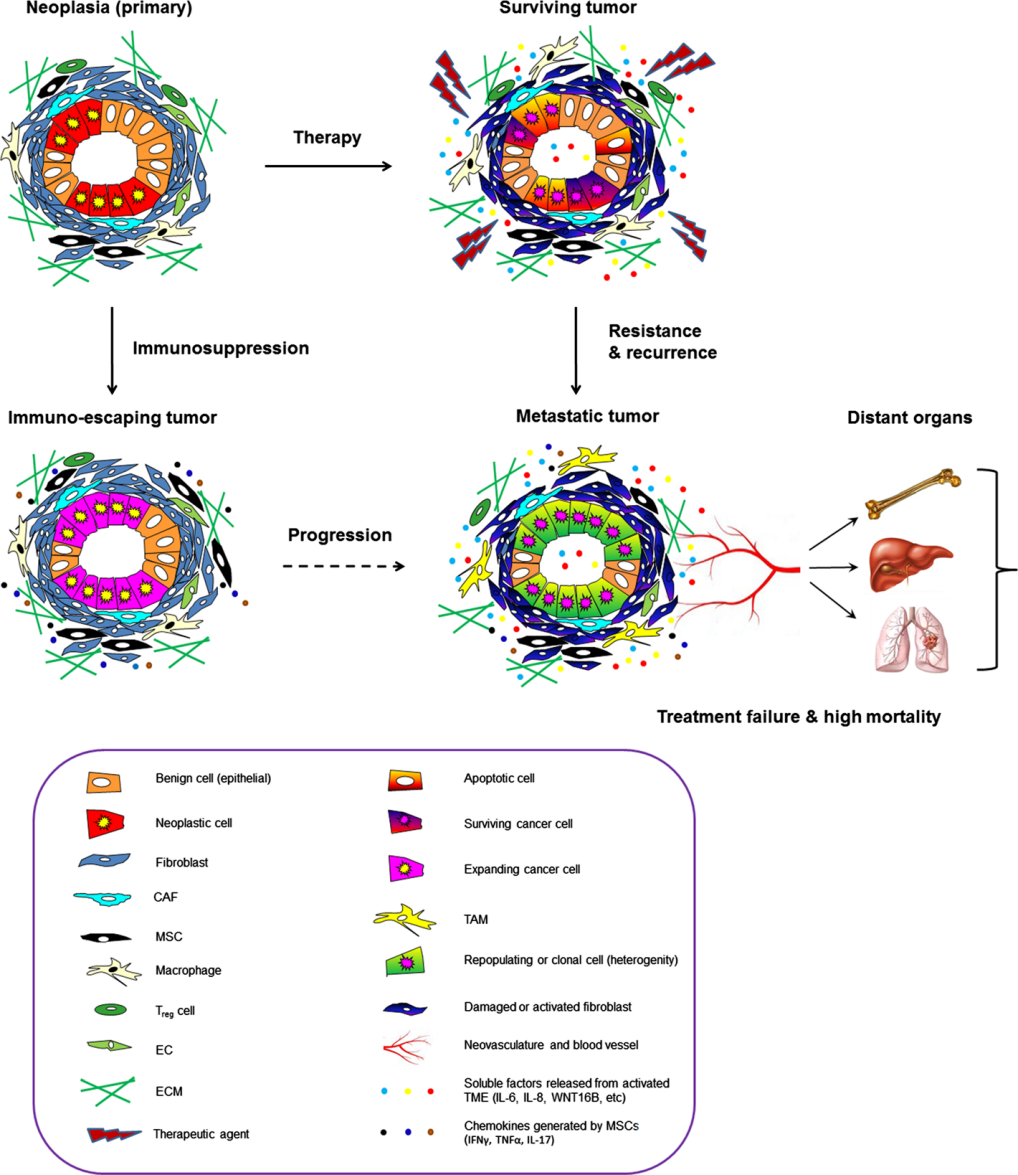
**Cancer develops in a complex and dynamic TME, which exerts profound impacts to disease progression.** Cancer cells are in close relationship with diverse non-cancer cell types within the TME, forming a functional nexus that facilitates tumor initiation, survival, and exacerbation. Cytotoxicity generated by treatments including chemotherapy, radiation, and targeted therapy eliminates many malignant cells within the cancer cell population; however, surviving cells are frequently retained in specific TME niches. Such protection minimizes the sensitivity to anti-cancer agents and generates resistant subclones through distinct mechanisms, prominently through acquired resistance conferred by a large body of soluble factors released from damaged or remodeled stroma. Alternatively, BMDCs, including MSCs and Tregs, mediate immunomodulation and prevent inflammation by restraining the activity of cytotoxic T cells, correlating with poor prognosis. Either acquired resistance or immunosurveillance evasion promotes cancer cell survival and subsequent expansion, allowing development of more advanced phenotypes, including tumor relapse, distant metastasis, and therapeutic failure, eventually causing high mortality in clinical settings. CAF, Carcinoma-associated fibroblast; MSC, Mesenchymal stem cell; BMDC, Bone marrow-derived cell; Treg cell, Regulatory T cell; EC, Endothelial cell; ECM, Extracellular matrix; TAM, Tumor-associated macrophage.

Altogether, it is increasingly evident that distinct stages of tumor advancing are subject to continuous and comprehensive influence of the TME in a special and temporal manner, underscoring the necessity to consider the TME in the context of clinical management (Figure 1).

## Therapeutic strategies to manipulate the TME with acumen

Innate resistance to clinical therapeutics is a hallmark of cancer; however, acquired resistance has also emerged as a daunting challenge to anticancer treatments by minimizing the efficacy of otherwise successful regimens. The vast majority of mainstay therapeutic strategies against human tumors are designed to target intrinsic traits of cancer cells. In contrast, stromal cells within the TME are generally stable in genetics and/or epigenetics and thus less likely to be susceptible to diverse mechanisms of therapeutic resistance. Further, given the accumulating evidence of overwhelming heterogeneity at each aspect of tumor evolution which is significantly subject to functional influence of cancer cell extrinsic compartments, targeting the TME turns out to be quite urgent and should be given enough priority [97-99].

In the tumor, continuous interactions between cancer cells and the surrounding TME actively occur via direct intercellular contact or through secreted signaling molecules. To date, a handful of targeted therapeutics against specific stromal compartments is successfully implemented, which shows decent promise in substantially minimizing pathological contributions of the TME in clinical settings (Table 2). Based on the growing data from clinical development and relevant trials, however, some anticancer drugs failed to show convincing benefits. For example, a group of pan-protease inhibitors, such as marimastat, tanomastat, and prinomastat, could not deliver significant therapeutic advantage over standard-of-care treatments, possibly due to the fact that MMP activities are more closely correlated with early stage tumors rather than late-stage malignancies [6]. Moreover, although mounting preclinical studies substantiated enhanced expression of Hedgehog ligands across multiple forms of cancer and associated stromal fibroblast activation, suppressors against the major Hedgehog pathway protein Smoothened, mainly vismodegib and saridegib, were unable to prove their efficacy except some limited and transient responses [100,101].

**Table 2:** **A representative panel of therapeutic agents that target specific compartments of TME, an occult culprit hiding in the backdrop of pathologies**

**Molecule**	**Target**	**Molecular type**	**Company**	**Status**
**ECM/fibroblasts**				
Sonidegib	SMO	Small molecule	Novartis	Phase II (NCT01708174, NCT01757327, NCT02195973)
**Vasculature**				
Bevacizumab	VEGFA	Antibody	Genentech/Roche	FDA-approved ((BLA) 125085)
Vandetanib	VEGFRs, PDGFRs, EGFR	Small molecule	AstraZeneca	FDA-approved ((NDA) 022405)
Sunitinib	VEGFRs, PDGFRs, FLT3, CSF1R	Small molecule	Pfizer	FDA-approved ((NDA) 021938)
Axitinib	VEGFRs, PDGFRs, KIT	Small molecule	Pfizer	FDA-approved ((NDA) 022324)
Sorafenib	VEGFRs, RAF PDGFRs, KIT	Small molecule	Bayer	FDA-approved ((NDA) 021923)
Pazopanib	VEGFRs, PDGFRs, KIT	Small molecule	GlaxoSmithKline	FDA-approved ((NDA) 022465)
Cabozantinib	VEGFR2, RETMET	Small molecule	Exelixis	FDA-approved ((NDA) 023756)
Ziv-aflibercept	VEGFA, VEGFB, PIGF	Receptor-Fc fusion	Regeneron	FDA-approved ((BLA) 125418)
AMG-386	ANG2	RP-Fc fusion protein	Amgen	Phase III (NCT01204749, NCT01493505, NCT01281254)
Parsatuzumab	EGFL-7	Antibody	Genentech/Roche	Phase II (NCT01399684, NCT01366131)
Enoticumab	DLL4	Antibody	Regeneron	Phase I (NCT00871559)
Demcizumab	DLL4	Antibody	OncoMed	Phase I (NCT00744562, NCT01189968, NCT01189942, NCT01189929)
Nesvacumab	ANG2	Antibody	Regeneron	Phase I (NCT01688960, NCT01271972)
**Immune**				
Ipilimumab	CTLA-4	Antibody	Bristol-Myers Squibb	FDA-approved ((BLA) 125377)
Sipuleucel-T	PAP	DC vaccine	Dendreon	FDA-approved ((BLA) 125197)
Aldesleukin	IL-2	RP	Prometheus	FDA-approved ((BLA) 103293)
IFN-α-2b	IFN-α receptor	RP	Merck	FDA-approved ((BLA) 103132)
MK-3475	PD1	Antibody	Merck	Phase III (NCT01866319)
Nivolumab	PD1	Antibody	Bristol-Myers Squibb	Phase III (NCT01642004, NCT01668784, NCT01673867, NCT01721746, NCT01721772, NCT01844505)
Nivolumab	OX40	Antibody	Bristol-Myers Squibb and PPMC	Phase III (NCT01642004, NCT01668784, NCT01673867, NCT01721746, NCT01721772, NCT01844505)
MPDL-3280A	PDL1	Antibody	Genentech/Roche	Phase II (NCT01846416)
PLX-3397	KIT, CSF1R, FLT3	Small molecule	Plexxikon	Phase II (NCT01349036)
BMS-663513	CD137 (4-1BB)	Antibody	Bristol-Myers Squibb	Phase II (NCT00612664)
Blinatumomab	CD3 and CD19	Bi-specific scFv	Amgen	Phase II (NCT01741792, NCT01466179, NCT01207388, NCT01471782, NCT00560794, NCT01209286)
AMG-820	CSF1R	Antibody	Amgen	Phase I (NCT01444404)
AMP-224	PD1	Antibody	GlaxoSmithKline	Phase I (NCT01352884)
TRX-518	GITR	Antibody	GITR, Inc.	Phase I (NCT01239134)
IMC-CS4	CSR1R	Antibody	ImClone/Eli Lilly	Phase I (NCT01346358)
CP-870,893	CD40	Antibody	Pfizer	Phase I (NCT00711191, NCT01008527, NCT00607048, NCT01456585, NCT01103635)

References listed in the status column pertain to the molecule as a TME-modifying agent, either the FDA application, where approved, or the national clinical trial identification of the oncology trial in the latest phase is listed (note that in some cases the drug may also be tested or approved for an indication for which it acts directly on the tumor cell compartment, which will not be referenced here). ANG2, Angiopoietin 2; BLA, Biological license application; CD40, Cluster of differentiation antigen 40; CD137, Cluster of differentiation antigen 137; CSF1R, Colony stimulating factor 1 receptor; CTLA-4, Cytotoxic T-lymphocyte-associated antigen 4; DC, Dendritic cell; DLL4, Delta-like 4; ECM, Extracellular matrix; EGFL-7, Epidermal growth factor like 7; EGFR, Epidermal growth factor receptor; Fc, Fragment, crystallizable; FDA, Food and Drug Administration; FLT3, Fms-like tyrosine kinase 3; GITR, Glucocorticoid-induced TNFR-related; IFN, Interferon; IL-2, Interleukin 2;KIT, Stem cell factor receptor; MET, Hepatocyte growth factor receptor; NCT, National clinical trial; NDA, New drug application; OX40, Cluster of differentiation antigen 134; PAP, Prostatic acid phosphatase; PD-1, Programmed death-1; PDGFR, Platelet-derived growth factor receptor; PDL1, Programmed death ligand 1; PIGF, Phosphatidylinositol-glycan biosynthesis class F protein; PPMC, Portland Providence Medical Center; RAF, Rapidly accelerated fibrosarcoma; RET, Rearranged during transfection; RP, Recombinant peptide; scFv, Single-chain Fv; SMO, Smoothened; VEGF, Vascular endothelial growth factor; VEGFR, Vascular endothelial growth factor receptor. Table adapted from reference [6] of this article (Junttila and de Sauvage) with permission from Nature, copyright 2013. Note, agents that either failed to be effective in clinical trials or have been officially terminated are removed from the current list.

**Figure 2:**
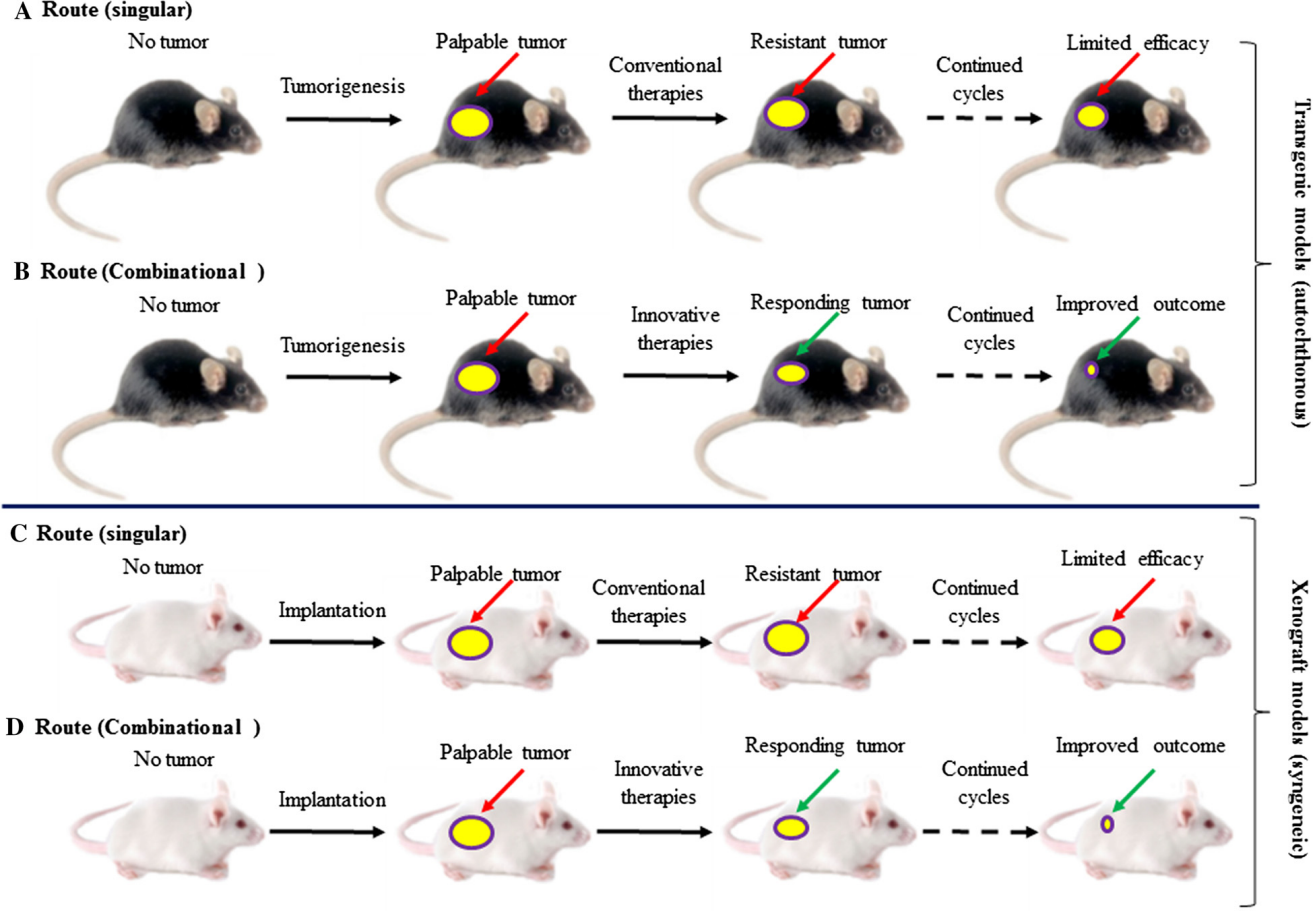
**Illustrative models for the preclinical evaluation of novel anticancer regimes that incorporate TME-targeting agents. (A)** Route 1 (singular), tumors develop in transgenic mice before the preclinical administration of chemotherapy or targeted therapy is applied as a singular agent. Dramatic cancer resistance is observed in such a therapeutic approach, with only limited efficacy available. **(B)** Route 2 (combinational), in contrast to route 1, an updated regime incorporating the novel agents (small molecule inhibitor or monoclonal antibodies) into the treatment program, which allows targeting both the tumor and TME. Significant disease regression follows after several cycles of the novel treatments, with much higher preclinical index achieved. **(C)** Route 3 (singular), tumors develop in the immunocompetent (wild type) mice xenografted with cancer cells and stromal cells from the same genetic and/or strain background as the host. Upon exposure to treatments as in Route A, a low outcome is observed. **(D)** Route 4 (combinational), tumors develop in the xenograft mice as in C, harboring implanted cancer and stromal components. Once receiving the same treatments as in Route B, animals present significantly improved therapeutic efficacy. (Note, in routes C and D, the preclinical paradigm in prospective trials exclude PDX, although it is a highly recommended model for many cancer studies).

To date, the mainstay of therapeutic strategies that target the TME *in vivo* has established translational avenues and paved the road for continued inputs into clinical frontiers. Despite the preliminary success of TME-targeted therapies, there remain several important issues that must be clearly addressed. Conventional anticancer treatments frequently cause structural and functional alterations of the TME, which contribute to acquired resistance and severely compromise clinical outcomes by generation of cancer-protective niches, emphasizing the necessity to consider the global TME response in future clinical intervention. First, novel biomarkers that indicate the treatment consequence and image the extent of TME damage through examination of patient bio-specimens (particularly serum samples) will allow for real time surveillance, therapeutic regimen optimization, and drug design innovation – each case is eagerly desired. Identification and selection of these molecules to establish a diagnostic panel applicable to clinical conditions would significantly accelerate the advancement of translational medicine. Second, the TME-stimulated cancer resistance and disease resilience may be technically prevented by rational administration of agents between therapeutic cycles to periodically retard key regulators of the TME signaling network, a feasible approach to minimize the influence of tumor-promoting factors from activated stromal cells that either develop a secretory phenotype or exert other adverse actions to enhance pathologies [5,102]. Third, MSCs are currently being tested in clinical trials for the treatment of various diseases owing to their potential and ability to differentiate into various cell lineages, including personalized treatments in regeneration medicine. However, immunosuppressive and pro-metastatic activities mediated by MSCs far outweigh their stemness-derived benefits, particularly in the case of cancer. Thus, the caveat is, although normal MSCs are crucial in wound healing and tissue remodeling, prospective therapies restraining tumor-associated MSCs hold the promise to improve the overall outcome of anticancer treatments. To this end, identification of biomarkers expressed by such a special subset of MSCs presents a new task before relevant targeting is available. Further understanding the nature and mechanisms of stromal cells, particularly BMDCs, in the physiological and malignant contexts will pave the road for new therapies against deleterious components of the TME, especially the immune-interfering partners. Last but not least, successful preclinical evaluation of combination therapies that target both tumor and adjacent TME requires inputs from effective experimental systems. Traditional *in vitro* cell culture studies meet major difficulty as specific elements of a typical TME, such as immune cells and vasculature, cannot be easily integrated. The recently emerging model of patient-derived xenograft reflects the complexity of tumors including the structural and functional heterogeneity of TME, but the host is immunodeficient [6]. In contrast, autochthonous or genetically engineered animals develop tumors that are initiated within the native environment and progress with an intact TME thereby engaging essential responses. In parallel, syngeneic models taking cancer and stromal cells derived from the same genetic background at orthotopic sites allow co-evolution of the tumor and nearby microenvironment, thus demonstrating significant efficacy for preclinical studies (Figure 2).

## Conclusions and future directions

Tumors evolve in a complex, dynamic, and functionally multifaceted microenvironment, which they rely upon for sustained growth, invasion, and metastasis. Unlike cancer cells, stromal populations within the TME are genetically stable, and thus represent an attractive therapeutic target with minimal risk of treatment resistance and disease relapse. TME-oriented research is increasingly encouraged and advocated, including the endeavors made in basic, clinical, and translational medicine. In such an exciting era of TME biology, experimental data have led to new scientific concepts and identified novel therapeutic targets to control the TME-related pathologies. However, there are not only major advances but daunting challenges, the latter including how to uncover and restrain susceptible nodes in the structurally complex and functionally intertwined TME system. Given that key signaling pathways frequently crosstalk and mutually interact in an intricate network, insights into how to solve the tortuous maze in a wider landscape and how tumor type-specific TMEs may respond differently to current standard-of-care therapies remain as important issues to tackle with intelligence. Fortunately, with the wealth of data accumulated so far, we now have a roadmap to convert these challenges into opportunities. For instance, when defining predictive markers that will eventually aid in the selection of patients who most likely benefit from intervention, analysis based on the entire TME is an essential step of utmost importance to determine specific therapies to employ [17,103,104]. To this end, gene expression profiling has been proposed as predictive for response to a given therapy, while in the coming years a panel of markers will become available to achieve the predicted goal. More importantly, cancer cell-directed agents should be combined with the TME-targeting therapies as it is increasingly clear that stromal cells modulate the efficacy of a broad range of standard chemotherapies and targeted agents. Last but not least, manipulating a dysfunctional TME is critical and will yield striking results in cancer prevention, pathological control, and disease remission, as evidenced by the recent success of multiple pilot trials in clinical oncology.

## Abbreviations

BMDC: Bone marrow-derived cell
BM-MSC: Bone marrow-derived MSC
CAF: Carcinoma associated fibroblast
CCL: Chemokine CC motif ligand
CSC: Cancer stem cell
CXCL: Chemokine CXC motif ligand
ECM: Extracellular matrix
EGF: Epidermal growth factor
EGFR: Epidermal growth factor receptor
EMT: Epithelial-mesenchymal transition
FGF: Fibroblast growth factor
IDO: Indoleamine 2,3-dioxygenase
IFN-γ: Interferon gamma
IGF-1: Insulin-like growth factor-I
IL: Interleukin
iNOS: inducible nitric oxide synthase
JAK2: Janus kinase 2
LPC: Leukemia propagating cell
MAPK: Mitogen activated protein kinase
MDSC: Myeloid-derived suppressor cell
MET: Mesenchymal-to-epithelial transition
miRNA: micro RNA
MMP: Matrix metalloproteinase
MSC: Mesenchymal stem cell
NK: Natural killer
NO: Nitric oxide
TAM: Tumor associated macrophage
TGFβ: Transforming growth factor beta
TME: Tumor microenvironment
TNF-α: Tumor necrosis factor-alpha
Treg: regulatory T cell
VEGF: Vascular endothelial growth factor
